# Recurrent Interstitial Pneumonitis in a Patient with Entero-Behçet's Disease Initially Treated with Mesalazine

**DOI:** 10.1155/2016/5636489

**Published:** 2016-06-26

**Authors:** Akihiro Nakamura, Tomoya Miyamura, Brian Wu, Eiichi Suematsu

**Affiliations:** ^1^Department of Internal Medicine and Rheumatology, Clinical Research Institute, National Hospital Organization, Kyushu Medical Center, 1-8-1 Jigyohama, Chuo-ku, Fukuoka 810-8563, Japan; ^2^Department of Genetics and Development, Krembil Research Institute, Toronto Western Hospital, 60 Leonard Avenue, Toronto, ON, Canada M5T 2S8

## Abstract

A 65-year-old man with entero-Behçet's disease (BD) being treated with mesalazine was presented to our hospital complaining of dyspnea. Computed tomography (CT) of the chest showed ground-glass opacities and he was initially diagnosed with mesalazine-induced interstitial pneumonitis (IP). Besides the discontinuation of mesalazine, a high dose of oral prednisolone was administered and the patient seemed to recover. However, four months later, dyspnea recurred and repeated CT revealed more extensive pulmonary infiltration despite steroid therapy. After the exclusion of infections, we suspected either a recurrence of mesalazine-induced IP or BD-related IP as a clinical manifestation of BD. The patient was treated with intravenous methylprednisolone and cyclophosphamide, followed by orally administered azathioprine, based on the assumption of underlying vasculitis. Thereafter, his condition improved. BD-related IP is an extremely rare condition with limited reports in the literature. Mesalazine-induced IP is also uncommon but the prognosis is generally good after discontinuation of mesalazine with or without steroid therapy. We discuss an extremely rare case, especially focusing on BD-related IP and mesalazine-induced IP as a potential cause of recurrent IP in a patient with entero-BD.

## 1. Introduction

Entero-Behçet's disease (BD) is a particular type of BD according to the diagnostic criteria of BD from the Japanese Ministry of Health, Labour and Welfare. It is characterized by intestinal aphthae, typically located at the ileocecal lesion, as a prominent manifestation leading to recurrent abdominal pain, diarrhea, and melena. Large aphthae may cause intestinal perforations which often require emergency surgery due to panperitonitis. Although the exact pathogenesis of entero-BD remains unclear, inflammation of vessels (vasculitis) is considered a main culprit of intestinal involvement in patients with BD.

Chronic interstitial lung disease (ILD) is characterized by diffuse lung interstitial wall inflammation, often resulting in severe pulmonary fibrosis and impaired gas exchange. Although pulmonary involvement is uncommon in patients with BD, pulmonary artery aneurysm (PAA), pulmonary infarction, hemorrhage, thrombosis, bronchiolitis, and pleurisy have been reported [1]. More than 200 cases of BD with pulmonary involvement have been reported in the literature [2]; however, interstitial pneumonitis (IP) associated with BD is extremely rare with few existing reports [3–8].

Aminosalicylates have been generally used in mild-to-moderate entero-BD. One of the serious side effects arising from the use of these agents is lung toxicity. Mesalazine is a newer drug with less frequent adverse events due to the lack of sulfapyridine moiety, but there have been rare reports of mesalazine-induced lung toxicity.

Here we report the case of recurrent IP in a patient with entero-BD initially treated with mesalazine. Despite the discontinuation of mesalazine and use of steroid, dyspnea persisted and computed tomography (CT) deteriorated. The patient's condition eventually improved on high dose oral prednisolone (PSL) in combination with monthly intravenous cyclophosphamide (IVCY) followed by azathioprine (AZA).

## 2. Case Report

**Figure 1:**
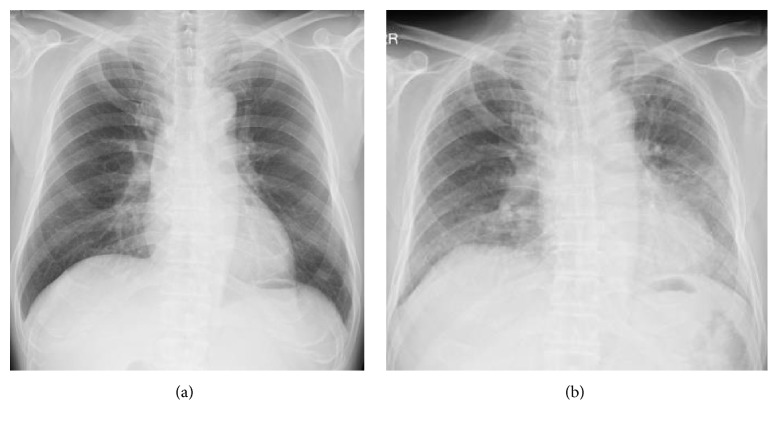
Chest X-ray images at the time of diagnosis with BD (a) and at the first time appearance of dyspnea (b).

A 55-year-old Japanese man was admitted for refractory stomatitis and persistent low-grade pyrexia. Four weeks prior to admission, he consulted a primary care physician and was diagnosed with acute bronchitis, but the fever persisted despite treatment with oral antibiotics. On admission, his symptoms had become more extensive and he was complaining of mild watery diarrhea without hematochezia. His vital signs were as follows: pulse of 75 beats per minute, blood pressure of 102/63 mmHg, respiratory rate of 16 breaths per minute, temperature of 38.0°C, and oxygen saturation of 97% on room air. Several areas of painful stomatitis were found in his mouth. On auscultation both lung fields were clear. Erythema nodosum was present on both lower limbs and arthritis was evident in bilateral knee and ankle joints. No genital ulcers were observed. Ophthalmologist did not detect any visual abnormalities including uveitis. Laboratory findings on admission revealed a white blood cell count of 13.4 × 10^9^/L with 86% of neutrophils, hemoglobin of 10.3 g/dL with a mean corpuscular volume of 99.6 fL, and reticulocyte count of 1.8%. Platelet count was within the normal range. Serum iron level was low (14 *μ*g/dL), unbound iron-binding capacity was normal (93 *μ*g/dL), and ferritin was elevated (619 ng/dL). Serum inflammatory markers were elevated, including C-reactive protein (85 mg/L), serum amyloid A (756 *μ*g/mL), and erythrocyte sedimentation rate (91 mm/h). Urinalysis revealed no significant findings. The result of HIV test was negative. HLA typing was positive for the B51 haplotype. Cultures of blood, urine, and sputum obtained on admission were sterile, and chest X-ray showed no significant findings (Figure 1(a)). An electrocardiogram revealed sinus rhythm at a rate of 68 beats per minute with normal intervals.

**Figure 2:**
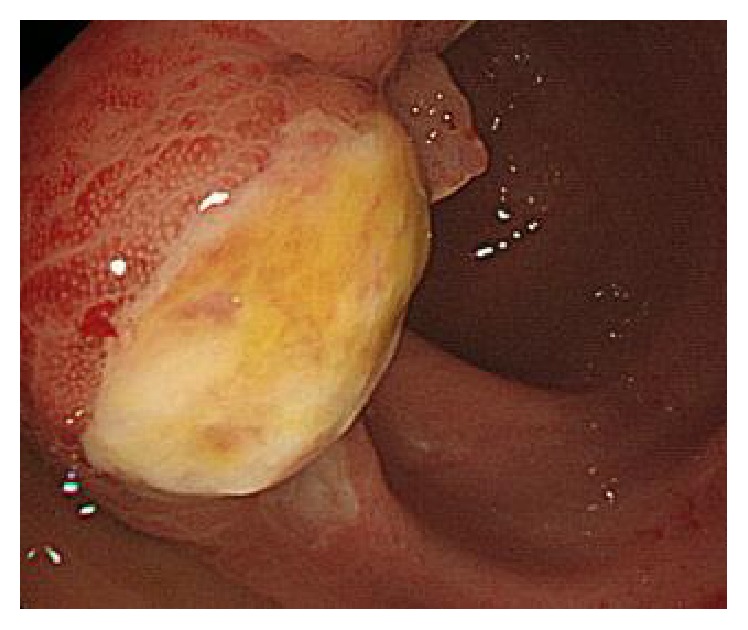
An ulcer on the ileum identified by colonoscopy.

On the fourteenth day of admission, the patient found watery diarrhea with abdominal discomfort. Colonoscopy revealed ulceration of the ileum, cecum, and ascending colon (Figure 2). An ileocecal biopsy showed mucosal atrophy and marked lymphocytic infiltration with a formation of lymph follicle. According to the criteria for BD from Ministry of Health, Labor and Welfare in Japan (chronic stomatitis, EN, arthralgia, and ileum ulcers), the patient was diagnosed with BD, specifically a type of entero-BD based on prominent intestinal manifestations. Treatment with prednisolone (PSL) (45 mg/day, 1 mg/kg/day), mesalazine (500 mg/day), and colchicine (1.0 mg/day) was initiated, and the symptoms of fever, arthralgia, and watery diarrhea subsided over the subsequent few weeks, and he was discharged.

**Figure 3:**
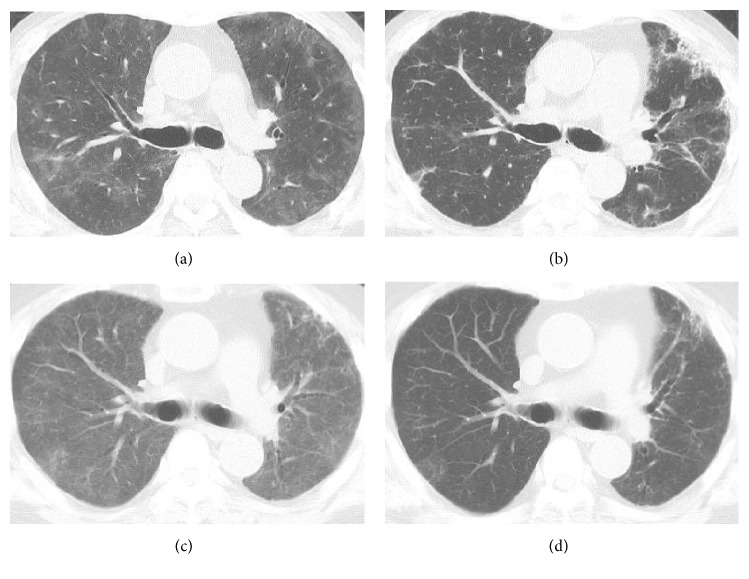
Computed tomography of the chest at the first time appearance of dyspnea (a), after treatment with prednisone 40 mg/day (b), at recurrence of dyspnea (c), and after treatment with methylprednisolone pulse and cyclophosphamide pulse therapy (d).

**Figure 4:**
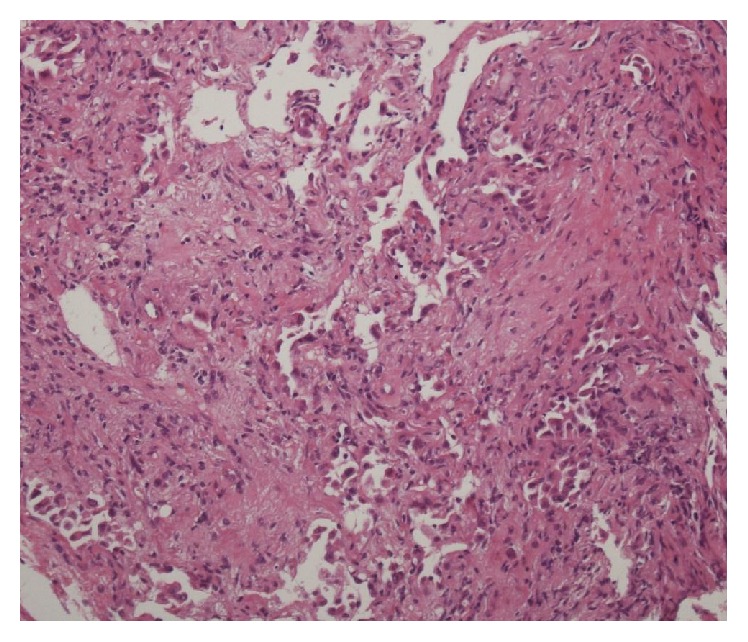
The result of transbronchial lung biopsy. Hematoxylin and eosin (HE) stain revealed diffuse lymphocytes infiltration without any findings of vasculitis (magnification: 100x).

Three months later, he was readmitted to our hospital complaining of subacute onset of exertional dyspnea, fever, and dry cough. Chest X-ray showed diffuse infiltrates (Figure 1(b)) and CT of the chest without contrast medium showed diffuse ground-glass opacities (GGO) in both lung fields (Figure 3(a)). We considered differential diagnoses of bacterial, viral, or fungal pneumonia, congestive cardiac failure, or IP associated with drug associated IP or BD activity. Laboratory data showed high levels of CRP (39.5) and ESR (66 mm/h) but no elevation of *β*-D glucan as well as negative results of cytomegalovirus (CMV) antigen (C7-HRP) and IgM antibody. We excluded congestive cardiac failure on the basis of an unremarkable cardiovascular examination, a normal result of brain natriuretic peptide (BNP), and essentially no dyskinetic movement of his heart assessed by echocardiography. Bronchoscopy, lavage, and culture found a predominance of CD8-positive T-lymphocytes over CD4-positive ones (CD4/CD8 ratio 0.62), with no bacterial growth. Additionally, polymerase chain reaction (PCR) for* Pneumocystis jirovecii *in bronchoalveolar lavage (BAL) was negative. Similarly, blood and sputum cultures were sterile. Transbronchial lung biopsy (TBLB) revealed nonspecific intestinal inflammation with fibrosis and no signs of vasculitis including lymphocyte infiltration around vessels or vascular occlusion (Figure 4). As a result of these findings, a drug-induced acute lung injury was suspected, with mesalazine as a potential trigger. Mesalazine was withdrawn and PSL increased to 40 mg/day (0.8 mg/kg). Thereafter, his symptoms and the GGO appearance on chest CT both improved compared with admission, although a small amount of fibrosis remained visible (Figure 3(b)). He was discharged after approximately 6 weeks in hospital and continued taking steroids.

**Figure 5:**
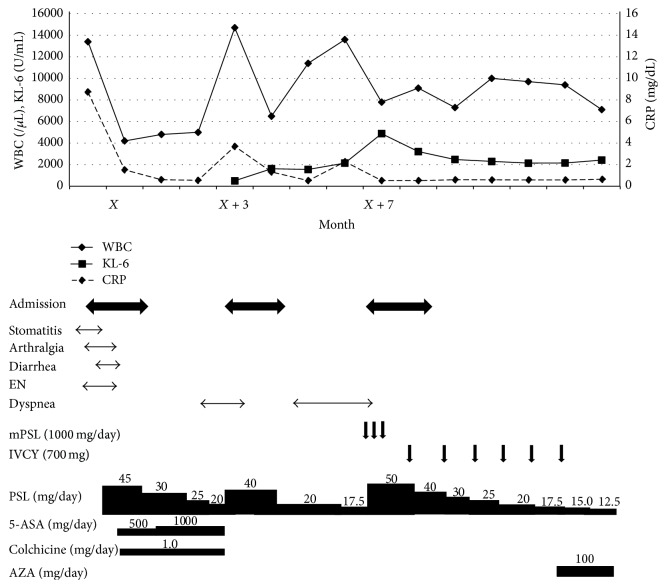
Clinical course of the patient. mPSL: methylprednisolone; IVCY: intravenous cyclophosphamide; 5-ASA: mesalazine; AZA: azathioprine; EN: erythema nodosum.

After another three months the patient was readmitted with worsening dyspnea. On examination, although body temperature and blood pressure were 37.5°C and 150/70 mmHg, respectively, respiratory rate was 28/min, and the oxygen saturation was 85% on room air. Chest CT without contrast revealed more widespread interstitial infiltration than the examination performed on the previous admission (Figure 3(c)). After exclusion of infections, we suspected either the diagnosis of persistent mesalazine-induced IP or IP secondary to BD. The patient was treated with a pulse of methylprednisolone (mPSL) 1 g/day for 3 days followed by PSL 50 mg/day orally and six doses of monthly IVCY (1000 mg/month). By the 7th day of admission, the symptoms improved and supplemental oxygen was no longer required. Since there was the possibility of BD-related IP despite steroid therapy, AZA was added to PSL as maintenance therapy. The appearance of unenhanced chest CT also improved (Figure 3(d)). He has since remained asymptomatic and the BD has been well controlled for 15 months to date (Figure 5).

## 3. Discussion

This is an extremely rare case of IP in a patient with entero-BD. Pulmonary findings have been reported in BD including PAA, pulmonary infarction, hemorrhage, and both organizing and eosinophilic pneumonias [1], but they are uncommon. There are several reports that have estimated the prevalence of pulmonary manifestations of BD to be between 1% and 7.7% [9, 10]. Among the pulmonary complications associated with BD, PAA is the most frequently occurring, as a consequence of vasculitis affecting the small pulmonary vessels [11]. The pulmonary arteries are the second most common site of arterial involvement after the aorta in patients with BD [2]. The interstitial pneumonia related to BD is also considered to be secondary to vasculitis of pulmonary vessels.

The most controversial point in the current case is whether the interstitial infiltration is due to BD or not: ILD associated with BD is certainly extremely rare. Furthermore, since there was no other systemic sign of BD at the time of respiratory dysfunction, it could be argued that BD was not the cause. However, only three months had passed since he was diagnosed with established entero-BD, so it was considered that the activity of BD might be still high. Particularly, as the central feature of entero-BD is vasculitis, it would not be surprising if vasculitis coexisted in lung and intestine. Furthermore, the IP occurred under the treatment of moderate dose of PSL (20 mg/day) and his condition responded well to immunosuppressant therapies including pulsed mPSL and IVCY, followed by PSL and AZA, demonstrating the existence of underlying vasculitis.

We also considered that mesalazine was a likely cause of his acute illness as lung damage is rarely seen soon after the drug has been administered [12–15], and TBLB showed nonspecific interstitial inflammation without any findings of vasculitis. In our case, the dyspnea began 3 months after the mesalazine had been started, and the finding of predominantly CD8-positive cells in the BAL was suggestive of drug-induced lung injury. However, dyspnea and more extensive infiltrates recurred three months after mesalazine had been withdrawn. In general, the mesalazine-induced lung injury only improves by discontinuing the drug. To our knowledge, there was no report that the immunosuppressants were needed in combination with steroid therapy to cure the mesalazine-induced IP. Although the efficacy of steroid therapy for mesalazine-induced IP is controversial, it may facilitate the recovery if discontinuing the drug is not sufficient [13].

We initially suspected the possibility of infection because the GGO and interstitial infiltration are often seen in patients with pneumonia caused by many pathogens.* Pneumocystis jirovecii*, fungi,* Cytomegalovirus*, and* Mycobacterium* are frequently implicated as major causative organisms in immunosuppressed patients with acute progressive GGO. However, none of these organisms were identified on BAL or sputum culture and the results of serum *β*-D glucan and cytomegalovirus antigen and IgM antibody were not detected. Taken together, the possibility of infection was excluded in this patient. Indeed, immunosuppressive therapy did not deteriorate the patient's condition.

It was true that bronchoscopy and TBLB were not performed on the third admission, since the patient's respiratory function was substantially compromised and he was hypoxemic. If we could identify some evidence of vasculitis, the making diagnosis would be easier. In previous reports (Table 1), pathological examination found that GGO and patchy infiltration like those seen in this case were consequences of organizing pneumonia or nonspecific IP. In these 6 previous reports, four reports described that the PAA coexisted with IP implying the presence of vasculitis; however, Ning and Nanke reported cases of IP in patients with BD without association with vasculitis as our case. The pathology on why interstitial pneumonia occurred in those patients has not been revealed, but it might be an omen of vasculitis.

Our first-line treatments were pulsed mPSL and IVCY for induction and PSL and AZA for maintenance therapy, based on the assumption at the time that the interstitial infiltration had been caused by vasculitis. Although the TBLB did not show the presence of vasculitis, the tissue obtained by TBLB was very tiny. Therefore, we considered that vasculitis could not be excluded. There have been reports of IP being caused by secondary vasculitis, which were also treated with IVCY followed by oral AZA [5–7]. One report states that parenchymal abnormalities including IP are usually associated with areas of infarction and lung hemorrhage caused by* in situ *thrombosis of pulmonary vessels and leaking aneurysms caused by vasculitis [9]. These may be forerunners of more serious complications, such as PAA formation. Histological examination of infiltrates is rarely performed, as an open approach would be warranted because of the risk of hemorrhage. Therefore the true incidence of interstitial lung involvement in BD may be underestimated.

In conclusion, this case is an exclusively rare case of recurrent IP in a patient with entero-BD. It is crucial to rule out the possibility of pulmonary infections and drug-induced lung injury when investigating the cause of IP, but BD-related interstitial infiltration caused by vasculitis should also be considered. Although pulmonary abnormalities are uncommon in patients with BD, the diffuse interstitial infiltration on both lungs in this case showed a good response to immunosuppressants including PSL, IVCY, and AZA. As relatively little is known about IP in BD, further research into the incidence and pathophysiology is needed.

## Figures and Tables

**Table 1:** Previous reports of interstitial pneumonia in patients with Behçet's disease and our case.

Author	Year published	Number of patients	CXR/CT findings	Pathological findings	Treatment
Efthimiou et al. [3]	1986	5	Opacities	Thrombosis, infarction, hemorrhage, fibrosis	N/A
Akoglu et al. [4]	1987	1	Diffuse reticulonodular infiltration	Interstitial pneumonia	PSL
Gül et al. [5]	1999	1	Mural thrombosis, GGO	Organizing pneumonia	mPSL pulse, IVCY monthly
Rutherford et al. [6]	2004	1	Patchy infiltration	Nonspecific interstitial pneumonia	PSL, IVCY monthly
Ning-Sheng et al. [7]	2004	1	GGO, multiple patchy consolidation	Organizing pneumonia	mPSL pulse, IVCY monthly
Nanke et al. [8]	2007	2	GGO, peripheral nodular opacities	Organizing pneumonia	PSL

Our case		1	GGO, patchy infiltration	Nonspecific interstitial inflammation	mPSL pulse, IVCY monthly

CXR: chest X-ray; GGO: ground-glass opacities; PSL: prednisolone; mPSL: methylprednisolone; IVCY: intravenous cyclophosphamide; N/A: not available.
